# Herausforderungen als Chance?

**DOI:** 10.1007/s00548-022-00792-4

**Published:** 2022-07-11

**Authors:** Martina Shakya, Kai Partale

**Affiliations:** 1 Hochschule Heilbronn, Heilbronn, Deutschland; 2 benchmark:services, Hofheim a. Ts., Deutschland

Die Coronapandemie ist nicht überwunden, da sieht sich die Welt durch den Ukrainekrieg mit einer neuen Krise und den daraus resultierenden wirtschaftlichen Schockwellen und Unsicherheiten konfrontiert. Auch der Klimawandel bedroht die Menschheit weiterhin, er wird die Tourismuswirtschaft auf lange Sicht beschäftigen.

Veränderte Ansprüche und Erwartungen von Reisenden in Bezug auf Nachhaltigkeit zeichnen sich nicht erst seit der COVID-19-Pandemie ab – trotz der empirisch nachgewiesenen „Attitude-Behaviour-Gap“, der Diskrepanz zwischen persönlicher Einstellung und der tatsächlichen Nachfrage nach umwelt- und sozialverträglichen Reiseangeboten. Die Tourismuswirtschaft, die etwa 8 % zu den globalen Treibhausgasemissionen beiträgt, muss nicht nur aktiven Klimaschutz betreiben, sondern sich auch an die vielfachen Folgen der globalen Erwärmung anpassen. In diesem Kontext taucht der Begriff der Resilienz, der Widerstandsfähigkeit, immer häufiger im tourismuswissenschaftlichen Diskurs auf. Während das Konzept der Nachhaltigkeit den Fokus auf Ressourcenbewahrung sowie intra- und intergenerationelle Gerechtigkeit legt, zielt Resilienz darauf ab, sich an ein zunehmend unsicheres Geschäftsumfeld anzupassen und flexibel auf Krisen reagieren zu können. Beide Konzepte ergänzen sich daher gegenseitig.

So stellt sich die Frage, ob und wie Destinationen und touristische Leistungsträger besser mit Herausforderungen umgehen können. Die Beiträge in diesem Themenheft zeigen exemplarische Lösungsansätze auf, beispielsweise durch einen Fokus auf die Bedürfnisse der eigenen Bevölkerung, auf eine veränderte Rolle des Destinationsmanagements oder die Erweiterung touristischer Angebote und Zielgruppen.

Der Tourismus ist und bleibt eine Schlüsselbranche – nicht nur in Deutschland, wo er bis zur Coronakrise mit einem Beschäftigtenanteil von 9 % deutlich relevanter war als die Automobilbranche. Vor allem für ärmere Länder birgt eine nachhaltige Tourismusentwicklung vielfältige Potenziale, die es zu nutzen und auszubauen gilt. Daher beziehen sich die Beiträge nicht nur auf Deutschland, sondern auch auf Länder des globalen Südens.

Die Pandemie zeigt, dass unsere global vernetzte Wirtschaft und unsere Hypermobilität maßgeblich zur Verbreitung des Coronavirus beigetragen haben. Reisen ist und bleibt jedoch auch und gerade in Krisenzeiten wichtig: Es erweitert den vielzitierten Horizont, treibt zu Innovationen und gegenseitigem Lernen an und fördert Toleranz und besseres Verständnis gegenüber fremden Kulturen und Sichtweisen. Der sächsische Bergbaubeamte Hanns Carl von Carlowitz ist dafür ein gutes Beispiel: Seine Erkenntnisse zu einer „nachhaltenden“ Forstwirtschaft gewann er auf seinen Reisen durch Europa im Rahmen der zu seiner Zeit üblichen „grand tour“ junger europäischer Adliger. Er hinterließ sie der Nachwelt in der 1713 niedergeschriebenen *Silvicultura oeconomica* und gilt seither als „Erfinder“ des Nachhaltigkeitskonzeptes.

Viel Freude beim Lesen wünschen

Martina Shakya

Kai Partale

